# Contrast-Enhanced Ultrasonography for Screening and Diagnosis of Hepatocellular Carcinoma: A Case Series and Review of the Literature

**DOI:** 10.3390/medicines7090051

**Published:** 2020-08-27

**Authors:** Kathryn L. McGillen, Syeda Zaidi, Amer Ahmed, Shantell Harter, Nelson S. Yee

**Affiliations:** 1 Department of Radiology, Penn State Health Milton S. Hershey Medical Center, Hershey, PA 17033, USA; sharter@pennstatehealth.psu.edu; 2 Stritch School of Medicine, Loyola University Chicago, 2160 S 1st Ave, Maywood, IL 60531, USA; szaidi2@luc.edu; 3 Chicago College of Osteopathic Medicine, Midwestern University, 555 31st St, Downers Grove, IL 60515, USA; aahmed77@midwestern.edu; 4 Division of Hematology-Oncology, Department of Medicine, Penn State Health Milton S. Hershey Medical Center, Next-Generation Therapies Program, Penn State Cancer Institute, Hershey, PA 17033, USA

**Keywords:** contrast-enhanced ultrasound, hepatocellular carcinoma, LI-RADS, chronic liver disease

## Abstract

**Background:** Contrast-enhanced ultrasound (CEUS) is a safe and noninvasive imaging technique that can characterize and evaluate liver lesions, and has been approved for this use in the Unites States since 2016. CEUS has been shown to be similar in accuracy to computed tomography (CT) and magnetic resonance imaging (MRI) for noninvasive diagnosis of hepatocellular carcinoma (HCC) and offers several advantages in certain patient populations who have contraindications for CT or MRI. However, CEUS has inherent limitations and has not been widely employed for evaluation of HCC. **Methods:** We present three retrospective cases of liver lesions in patients with cirrhosis, who underwent screening for HCC using concurrent, well-timed CT and CEUS. **Results:** In these cases, the liver lesions were better visualized and then diagnosed as malignancy via CEUS, whereas the lesions were best appreciated on CT only in retrospect. **Conclusions:** In some cirrhotic patients, a focal lesion may be more easily identifiable via CEUS than on CT and thus accurately characterized, suggesting an important and complementary role of CEUS with CT or MRI. Further studies are indicated to support the use of CEUS for the diagnosis and characterization of liver lesions in screening patients at risk for developing HCC.

## 1. Introduction

Hepatocellular carcinoma (HCC) is the most common primary liver tumor and the fourth most common cause of cancer-related mortality worldwide [1]. Chronic hepatic injury and liver cirrhosis are known to pose an increased risk for development of HCC over time [2]. Major risk factors for chronic hepatic injury include alcoholism, infection with hepatitis B or C virus, and nonalcoholic fatty liver disease [1]. Nonmodifiable risk factors include autoimmune hepatitis, Wilson’s disease, hereditary hemochromatosis, primary biliary cirrhosis, and alpha 1-antitrypsin deficiency [3]. The American Association for the Study of Liver Diseases (AASLD) and European Association for the Study of the Liver (EASL) guidelines recommend surveillance in patients at high risk for developing HCC. The goal is to detect HCC early when it is amenable to curative therapy such as liver transplantation or surgical resection, and to reduce mortality [4]. Otherwise, palliative liver-directed therapy, such as chemoembolization, radiofrequency ablation, and stereotactic body radiation therapy, is an option for treatment [5,6,7]. However, for patients with advanced or metastatic HCC, palliative systemic treatment is the only therapeutic option, and the associated survival benefit is limited [8,9,10].

HCC is commonly diagnosed by imaging studies including multiphase computed tomography (CT) or magnetic resonance imaging (MRI) scans with and without intravenous (IV) contrast. These are considered the gold standard imaging modalities for noninvasive diagnosis of HCC [11]. However, there are various limitations of CT and MRI scans for patients with HCC. For CT scans, contrast-related allergy, respiratory motion, renal impairment, and cumulative radiation doses, especially in young patients, are factors of concern. For MRI scans, respiratory motion, certain cardiac pacemakers or metal in the body, significant ascites, and claustrophobia may limit its utility for patients. Ultrasonography (US) is often used for screening, sometimes nearly exclusively, other times alternating with CT or MRI, the choice of which may depend upon the individual patient’s risk factors, clinician’s preference, and health insurance approval of advanced imaging. If a lesion is found on screening US, further characterization of the lesion with a contrast-enhanced examination is required. In recent years, contrast-enhanced ultrasonography (CEUS) has been utilized for diagnostic imaging evaluation of HCC in the United States, with a meta-analysis showing a sensitivity of 85% and specificity of 91% [12]. CEUS may provide unique advantages that complement conventional CT and MRI scans in patients at risk for HCC.

CEUS is an imaging modality that has been approved for use in the characterization of liver lesions in the United States since 2016. It utilizes the standard US techniques of grayscale and color Doppler, with the addition of an US-specific intravenous contrast agent. By enhancing the organs and targeted lesions, the contrast agent yields additional diagnostic information. Several brands are approved by the Food and Drug Administration in the United States, and they utilize similar microbubble technology. Of the agents approved, contrast with a sulfur hexafluoride core (Lumason^®^, Bracco Diagnostics Inc., Monroe Township, NJ, USA) is widely used in the United States, Europe, and China [13] and at our own institution. CEUS has several unique features in comparison with CT or MRI with contrast. The contrast agent used with US is made of microbubbles that are smaller than a red blood cell, so that they do not cross the endothelium but instead remain in the intravascular space [13,14]. The microbubbles burst after several minutes of imaging, with byproducts exhaled through the pulmonary system [13]. This is unlike the contrast agents used with CT or MRI scans, which diffuse into the parenchyma and are excreted through the kidneys (and sometimes biliary system) and therefore can be affected by poor hepatic and renal function. Because of this, CEUS agents are safe to use in patients with poor renal function. Additionally, because of their limited duration in the bloodstream, patients can be dosed several times in the same setting, as opposed to CT or MRI, for which a wait time of hours or even a day may be necessary to perform a second injection and repeat imaging. This allows evaluation of multiple lesions using CEUS in one setting, and more importantly, re-evaluation of a single lesion, if necessary.

For characterization of individual lesions within the liver, CEUS has been proven to have accuracy similar to that of CT or MRI [15]. The American College of Radiology (ACR) created the Liver Reporting and Data System (LI-RADS) for noninvasive imaging diagnosis of HCC in patients at risk for its development. ACR released LI-RADS criteria for both routine US and CEUS, with the latter utilizing the same numbering system as CT and MRI for noninvasively diagnosing HCC [16]. This diagnostic algorithm categorizes imaging findings, lesion size, and enhancement patterns on a scale of 1 to 5 [17]. LI-RADS 1–3 lesions range from benign to intermediate, while LI-RADS 4 and 5 lesions are likely and almost definitively HCC, respectively [18], and they can be diagnosed via imaging criteria alone. An additional category, LI-RADS M, denotes the presence of a malignant liver lesion, but this is not specific for HCC [19,20]. In at-risk patients, screening programs to detect early HCC, while it is treatable, are most often conducted with multiphase CT or MRI, but may be performed with US, although it is less sensitive [15]. If a suspicious area is identified on US, the patient will need to return and have a CT or MRI for further characterization, requiring two visits and creating additional medical bills. Within our practice, insurance companies have increasingly denied pre-authorization for the gold standard four-phase CT or MRI for screening of HCC. An alternative solution is to screen patients with CEUS. Moreover, as mentioned above, some patients have poor renal function or history of allergic reaction to contrast agents used with CT or MRI, and may be precluded from safe use of these tests. CEUS, therefore, has an important potential role in this population in particular.

While CEUS and CT/MRI share similar fundamental imaging diagnostic criteria via LI-RADS, there are still notable differences with regard to image acquisition, contrast material, and visualization [17]. Here, we present three patients who were undergoing routine screening for HCC due to chronic liver disease and found to have lesions present on grayscale ultrasound, and then proceeded immediately to CEUS. In this case series, we compare the visibility of liver lesions using CEUS against the gold standard four-phase CT. We present evidence that demonstrates the accuracy of CEUS for identifying malignancy in at-risk cirrhotic patients, suggesting an important and complementary role of CEUS with CT or MRI.

## 2. Materials and Methods

This is a retrospective review examining the electronic medical records and images at our 548-bed semirural institution, which is also a liver transplant center, as well as the pertinent literature about CEUS in HCC diagnosis. All CEUS were performed on a Siemens S2000 with 2.5 mL of Lumason^®^ IV contrast. All CT scans were performed on a 64-slice scanner utilizing 150 mL of Omnipaque™ 350 IV contrast unless otherwise stated—each scan consisted of noncontrast, late arterial phase (bolus-triggered), portal venous, and 3 min delayed phase imaging. None of our patients had MRI performed for liver imaging. We have obtained the written informed consent of patients. The Human Subjects Protection Office of the Penn State Health Milton S. Hershey Medical Center determined that this case study does not meet the definition of human subject research as defined in 45 CFR 46.102(d) and/or (f); Institutional Review Board (IRB) review and approval is not required.

## 3. Case Presentation

### 3.1. Case 1

A 62-year-old Caucasian man with advanced hepatic fibrosis secondary to hepatitis C viral infection was scheduled for a four-phase CT to screen for HCC, but was denied insurance pre-approval for the imaging study. He was diagnosed with hepatitis C viral infection a decade earlier, which was subsequently treated and cleared with interferon and ribavirin. Cirrhosis was confirmed via biopsy at that time, and stage III fibrosis diagnosed via biopsy four year later. At the time of his screening request, he had no additional medical problems or sequela of advanced liver disease. His physical exam was essentially normal—including vital signs and for stigmata of chronic liver disease. His alpha-fetoprotein (AFP) was 3.0 ng/mL with normal liver function tests, albumin, and platelets.

**Figure 1:**
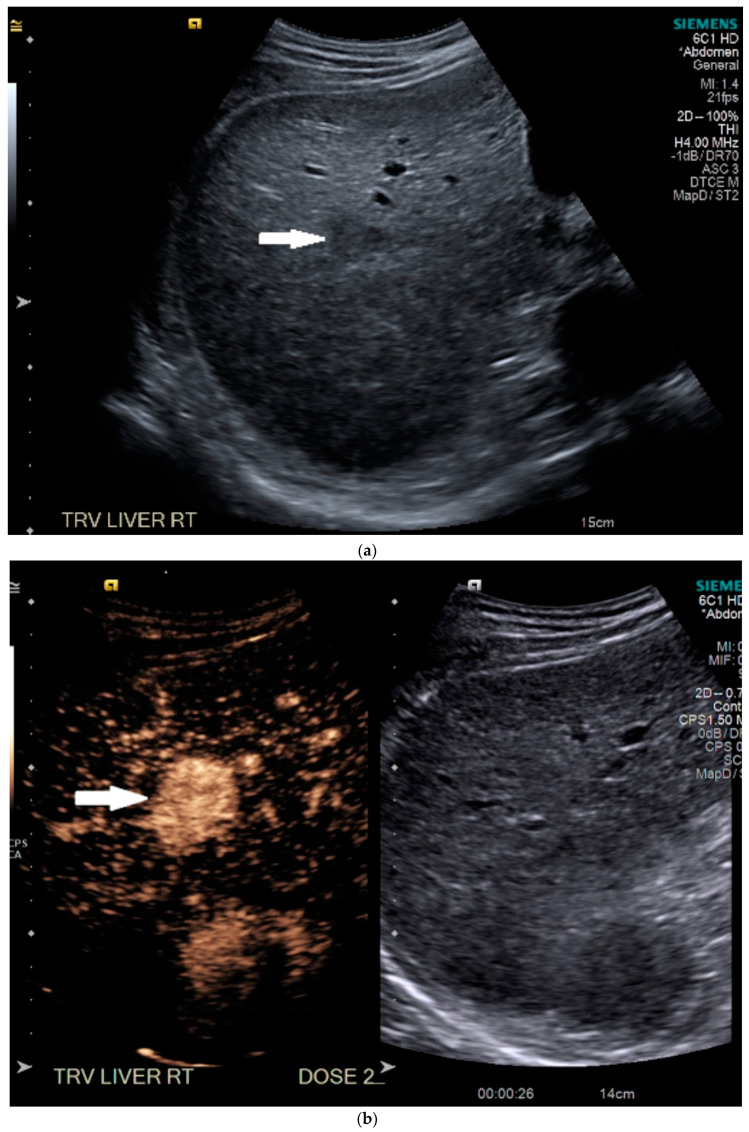

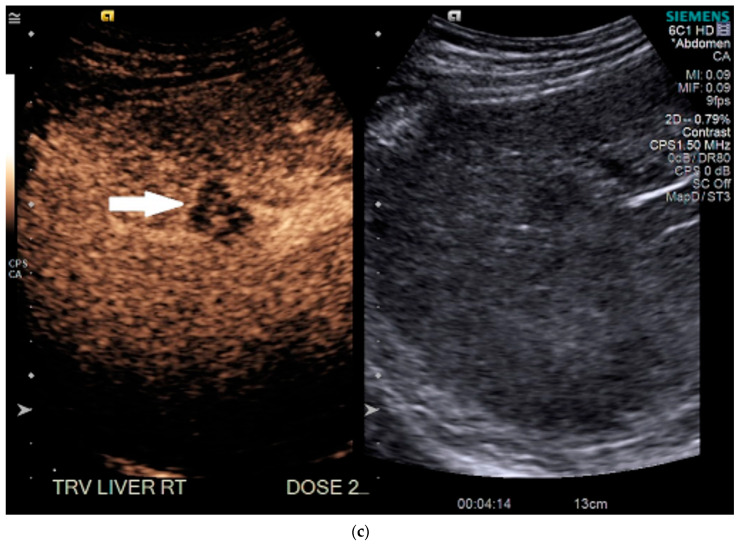
Patient 1 ultrasound images, with liver lesion denoted by white arrow: (**a**) grayscale images showing the hypoechoic lesion. Post contrast administration ultrasound in arterial phase with matched low-mechanical-index B-mode grayscale on the right and subtraction-type post contrast images on the left (**b**) and delayed phase (**c**) of the lesion, which shows marked washout. Liver Reporting and Data System (LI-RADS M).

**Figure 2:**
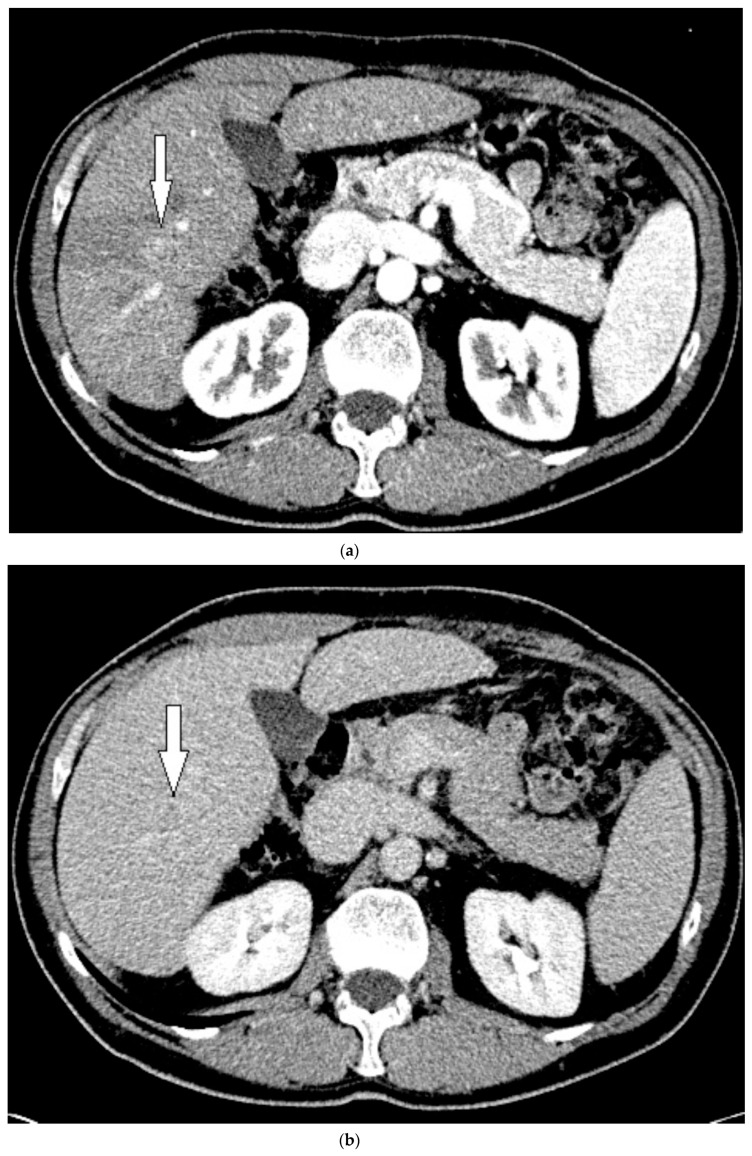
Patient 1 CT images using liver windows to highlight the subtle lesion easily seen on contrast-enhanced ultrasound (CEUS) (white arrow): (**a**) late arterial phase axial contrast-enhanced CT; (**b**) 3 min delayed phase.

Routine US showed a heterogeneous, coarsened liver parenchyma with a suspected 2.2 cm hypoechoic structure in the right lobe on a background of geographic hepatic steatosis (Figure 1). Contrast was then administered intravenously with targeted imaging of the lesion to determine if it was focal fatty sparing or tumor. The lesion markedly enhanced (Supplementary Video S1) and began washout rapidly, and on delayed imaging, showed intense washout. It was deemed a LI-RADS M because of its early enhancement and for its marked washout (Figure 1). Given the LI-RADS M designation and for treatment planning, a four-phase CT was then approved by his health insurance company. Despite its size, the lesion was poorly visualized on the well-timed late arterial phase (Figure 2). It was equally subtle on the portal venous and 3 min phases. Knowing where the lesion was on CEUS allowed the radiologist to confirm the findings on CT, where it was deemed a LI-RADS 5 lesion. However, without the pre-existing knowledge of its presence in that location, the lesion was initially missed. The late arterial phase on the CT was bolus-triggered, which resulted in scanning occurring at 35 s post injection. Interestingly, on the CEUS, the lesion was already washing out and was iso-intense to liver by 35 s. This may explain why it was poorly seen on CT despite its intense, avid enhancement on US.

Given the LI-RADS 5 designation on CT, the lesion was not biopsied prior to treatment, as may be indicated by an initial LI-RADS M diagnosis. Instead, it was treated as HCC with several transcatheter arterial chemoembolizations (TACEs). Despite two treatments with TACE, persistent and progression of tumor was identified on follow-up CTs over the next 8 months.

### 3.2. Case 2

A 63-year-old Caucasian man with hepatic cirrhosis presented for screening US. He had been diagnosed with viral hepatitis C-induced cirrhosis and achieved a sustained virologic response after treatment with Vosevi. He had a well-compensated cirrhosis, with grade 1 esophageal varices and portal hypertensive gastropathy. Just prior to his US, he was asymptomatic. His physical examination was significant for hypertension, elevated body mass index (BMI) of 38, but with no physical signs of chronic liver disease. His albumin was low (3.3 gm/dL), as was his total protein (6.1 gm/dL). Liver function tests were normal except for an elevated total and direct bilirubin (4.4 and 0.8 mg/dL, respectively). His AFP level was normal at 5.7 ng/mL, and his hepatitis C viral load was undetectable.

**Figure 3:**
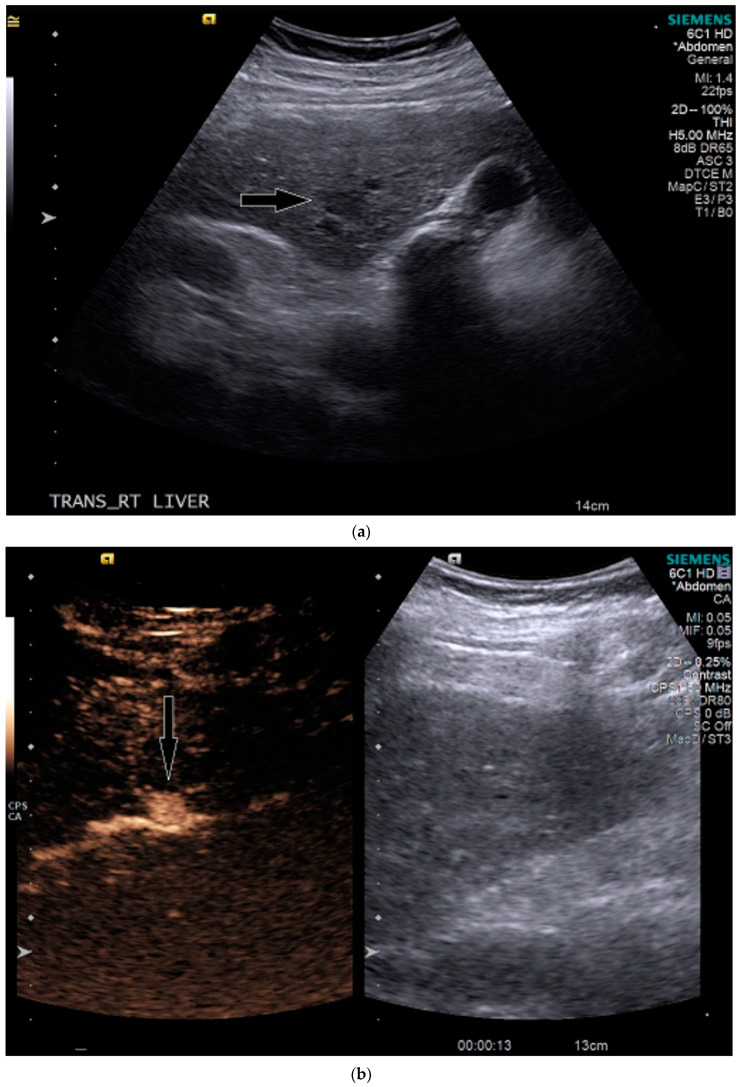

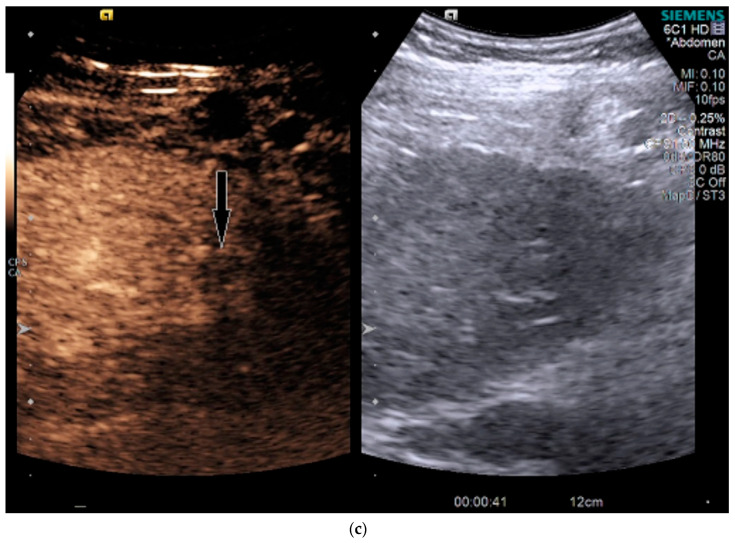
Patient 2 ultrasound images, with liver lesion denoted by black arrow: (**a**) grayscale images showing the hypoechoic lesion. Post contrast administration ultrasound in arterial phase (**b**) and early washout (**c**) of the lesion, LI-RADS M.

**Figure 4:**
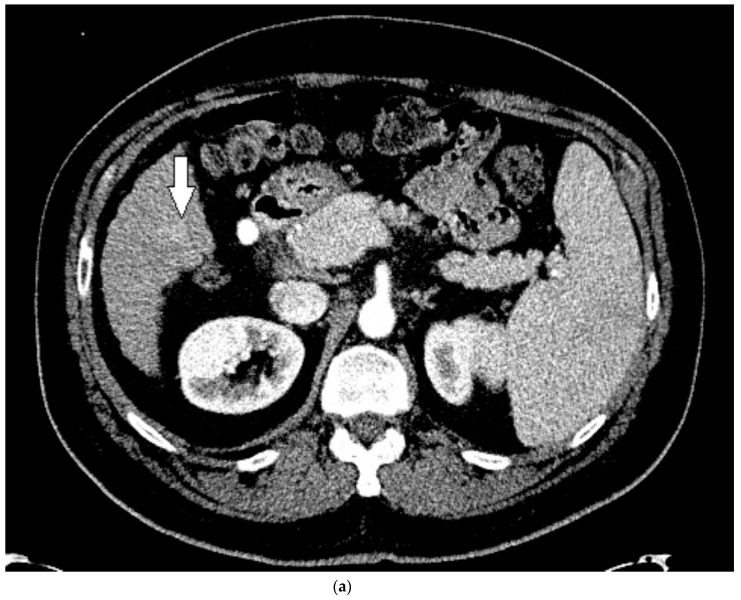

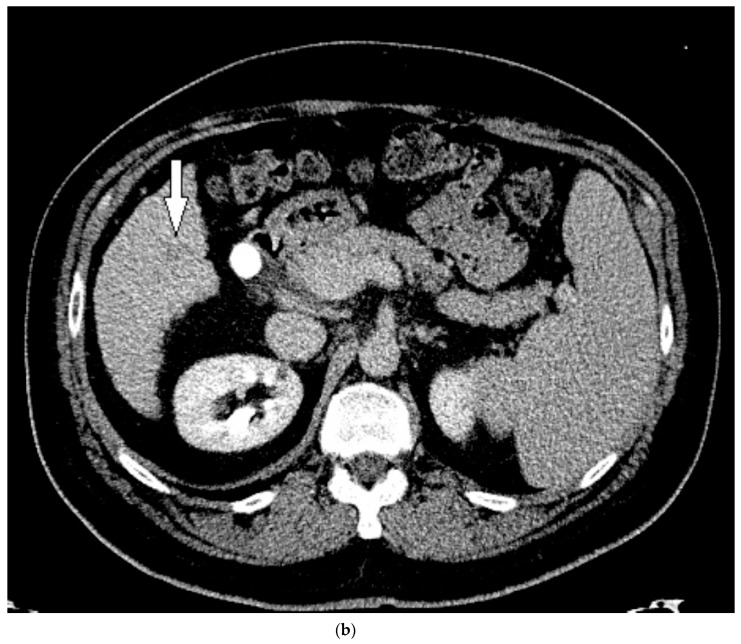
Patient 2 CT images using liver windows to highlight the lesion seen on CEUS (white arrow): (**a**) late arterial phase axial contrast-enhanced CT; (**b**) 3 min delayed phase.

On grayscale US, a subtle 1.2 cm hypoechoic focal area was identified on a background of echogenic, heterogeneous liver echotexture (Figure 3). This area was targeted with contrast, which showed avid arterial-phase enhancement, and rapid, but mild washout (Figure 3). This was also designated LI-RADS M due to the early washout. CT was performed to confirm the finding and initially, the lesion was not identified by the radiologist, despite a diagnostic late arterial phase. On closer inspection with the US finding in mind, subtle enhancement and washout on the CT was seen and considered LI-RADS 4 (Figure 4), and due to this designation and its small size, a shorter interval follow-up was performed at 3 months rather than 6 months.

The patient indeed followed up with an additional four-phase CT 3 months later, and the lesion had grown and was then clearly evident (Figure 4). It was upgraded to a LI-RADS 5, and the patient proceeded to treatment with a successful TACE. However, follow-up CTs over the next 6 months demonstrated development of new LI-RADS 5 lesions, which were subsequently treated with an additional TACE.

### 3.3. Case 3

A 59-year-old Caucasian man with hepatic cirrhosis secondary to alcoholism presented to the hospital with acutely worsening hepatic decompensation and acute on chronic renal dysfunction. He presented through the emergency department with worsening confusion and abdominal distention. He had a history of hepatic encephalopathy, moderate antral erosive gastritis, and ascites. At the time of admission, his physical examination was remarkable for scleral icterus, and fluid wave present in the abdomen. He was awake and oriented to person and place. Laboratory values were notable for an elevated, baseline international normalized ratio (INR) of prothrombin time (2.4), mildly elevated potassium (5.3 mmol/L), elevated creatinine (3.27 mg/dL, up from baseline of 2.3), platelets of 101,000/μL, and elevated liver function tests (alanine aminotransferase 57 unit/L, total bilirubin 10.3 mg/dL, alkaline phosphatase 185 unit/L) and ammonia elevated at 64 μmol/L. A four-phase CT had been performed 2 months earlier and interpreted as negative for HCC. US was performed to detect any HCC during acute evaluation for liver transplantation, in order to ensure the patient remained within transplant criteria.

**Figure 5:**
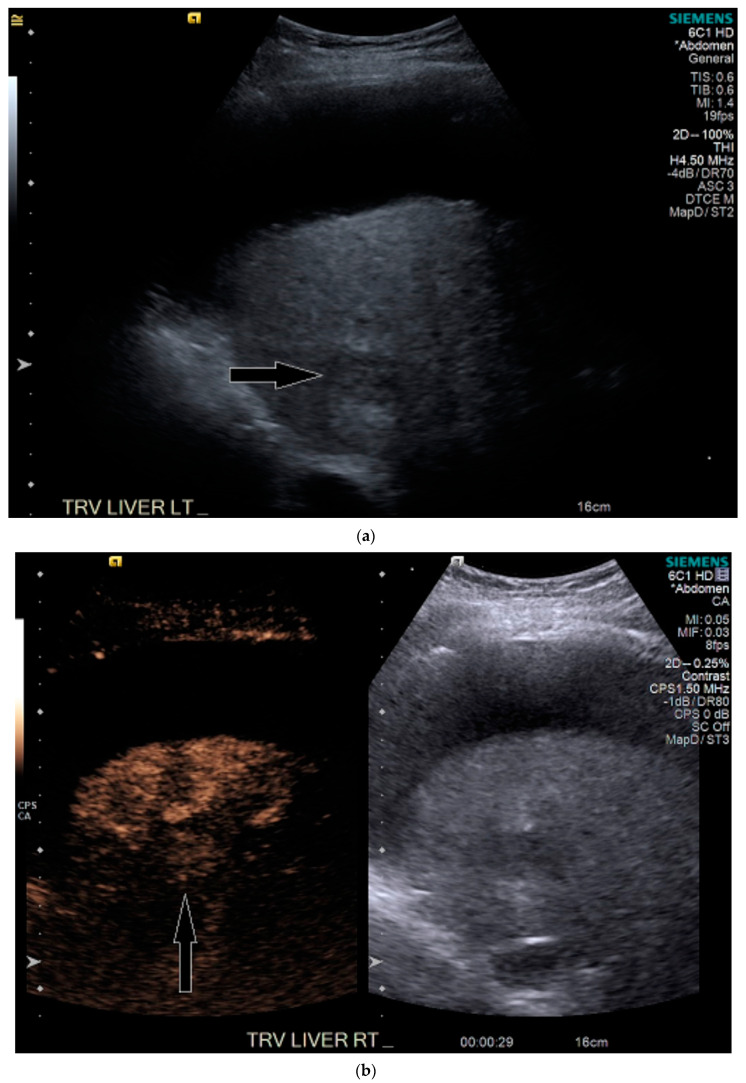

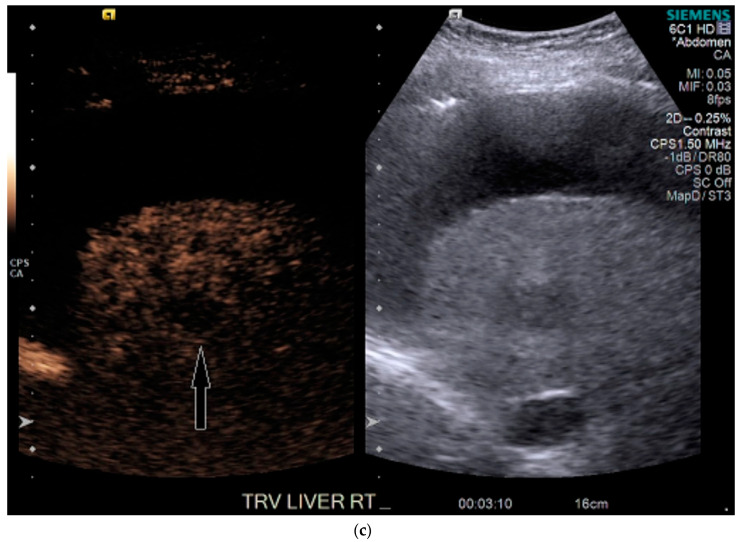
Patient 3 ultrasound images, ascites is present with lesion denoted by white arrow: (**a**) grayscale images showing the hypoechoic lesion. Post contrast administration ultrasound in arterial phase shows subtle enhancement compared with liver parenchyma at the same level (**b**) and delayed phase mild washout (**c**) of the lesion, LI-RADS 5.

On grayscale imaging, a 2.9 cm hypoechoic lesion at the dome of the right lobe on a background of heterogeneous liver parenchyma was identified (Figure 5). Due to his acute on chronic renal failure, CEUS was then performed, which showed subtle enhancement of the lesion, above background enhancement of the adjacent liver at the same depth. At or greater than 10 cm depth is most often the limit of penetration in standard CEUS software packages [13], but can be extended in the presence of ascites, as in our case. Subtle washout was identified on delayed imaging, and the lesion was deemed LI-RADS 5 (Figure 5).

**Figure 6:**
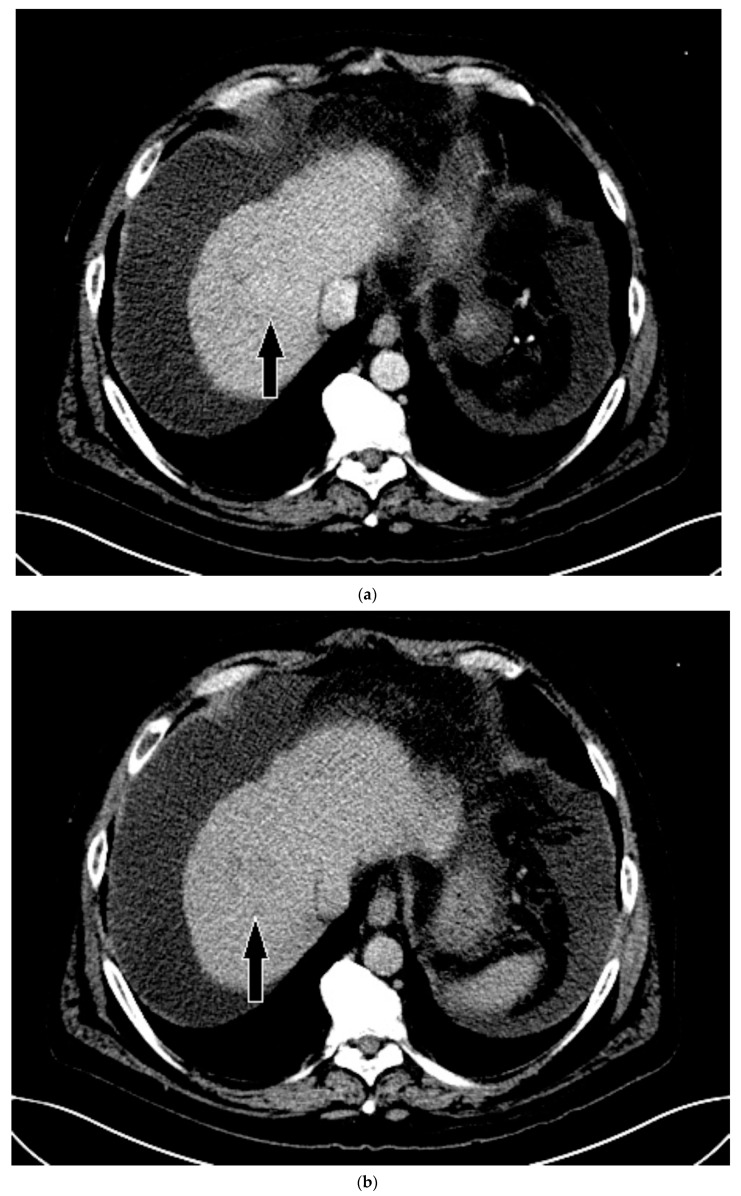
Patient 3 CT images using liver windows to highlight the subtle enhancement and washout of the lesion seen on CEUS (white arrow): (**a**) late arterial phase axial contrast-enhanced CT; (**b**) 3 min delayed phase.

His liver function continued to decline during his stay, and within two weeks he received a deceased donor liver transplantation. HCC within the explanted liver was confirmed by histopathological examination, corresponding to the findings on CEUS. Upon review of the prior CT, for which a reduced contrast bolus (75 mL Omnipaque™ 350) was given due to his chronic renal disease, the tumor was subtle but faintly visible with the knowledge of its location based on the findings on CEUS (Figure 6).

## 4. Discussion

With CEUS, patients at risk for HCC do not necessarily need to return for a second visit to get a CT or MRI when screening ultrasound identifies a focal abnormality—the lesion can be characterized at the time of the initial study. As of the 2017 edition of LI-RADS for CEUS [20], the United Network for Organ Sharing does not officially recognize CEUS, therefore while it can be used for transplant candidates, the patient will need a multiphase CT or MRI or biopsy to receive exception points for HCC. This is not a factor for patients who do not qualify for transplantation or are already at the top of the list. In our geographic area, MRI is not as commonly performed as CT for screening of HCC. Health insurance companies have increasingly denied screening CT, and so many of our patients are being monitored with US. However, even with characterization via CEUS, CT or MRI may still be performed subsequently for several reasons. In a practice just starting a CEUS program, it is useful to clinicians and radiologists to confirm that the diagnosis is correct via gold standard, noninvasive means. If a large lesion is confirmed as HCC, CT may still be required for staging purposes or for mapping arterial anatomy prior to TACE or transplant, however less phases may be required, resulting in decreased radiation. If the US is limited in visualizing the entire liver, CT or MRI may be appropriate to identify any additional lesions prior to treatment or transplant.

In the three presented cases, CEUS readily and accurately identified malignant lesions in each patient, as compared with their CT counterparts. For the first two cases, the patients had subsequent well-timed four-phase CTs, with very subtle lesions that may not have been detected without prior knowledge of the US findings. We postulate that this may have occurred in these cases, both of which were LI-RADS M, because the lesions enhanced so rapidly—likely earlier than the diagnostic late arterial phase CT—and then quickly became isoechoic, about the time the CT arterial bolus would have begun scanning (confirmed in our first patient). LI-RADS M lesions per ACR are diagnostic for a malignancy but are not specific to HCC [17,18]. HCC classically enhances in the arterial phase, and it has a late, but mild washout, which necessitates the timing for the CT phases, but in CEUS, continuous scanning can be performed. Those in the “M” category have rapid (less than 60 s) and/or marked washout on delayed images [20]. It is currently unknown how many “M” lesions represent HCC in at-risk patients diagnosed via CEUS, versus intrahepatic cholangiocarcinoma or mixed HCC–cholangiocarcinoma subtypes. However, it has been shown that LI-RADS M lesions on CT and MRI are HCC approximately one-third of the time [18]. It is unclear if this will hold true for CEUS, as it may identify “M” lesions at smaller sizes and earlier than CT, and thus if biopsied, could have a different, earlier tumor biology. In our first case, despite its appearance on CEUS, the tumor did not respond well to TACE. As a multiphase CT LI-RADS 5 designation is considered noninvasively diagnostic of HCC, a tissue biopsy would not be necessary. It is feasible that it could have represented a tumor that was not as responsive to TACE, and biopsy should be considered when CEUS and CT present discordant diagnoses of malignancy.

In our experience, CEUS identifies more “M” lesions than CT. In fact, both of our presented first and second cases were considered LI-RADS 4 or 5 by CT, despite the CEUS designation. Additional research into this area is needed—both in terms of histopathology of “M” lesions on CEUS in patients at risk for HCC, as well as if “M” lesions are more apparent and presenting earlier on CEUS than they are on well-timed CT. It is possible that MRI may have clearly identified both lesions when CT did not, as it does not rely solely on post contrast imaging. In the case of our second patient, a very small lesion may still be obscured via MRI if the patient is not able to consistently breath-hold, which is not usually an issue in CEUS.

In our third case, the lesion was not seen on the prior CT. This may have been due to location, and a lower dose of intravenous contrast that was used due to the patient’s impaired renal function. Interestingly, this lesion was well seen with CEUS, despite its challenging location at the dome, which can be a blind spot in US. We postulate that the ascites may have aided the CEUS exam—even though it increased the depth of the lesion, the ascites created a window to view the lesion, even with patient breathing, both of which would have negatively impacted on MRI.

HCC is the most common primary liver tumor [1], and imaging is a necessary component in screening patients at high risk of developing HCC with the current noninvasive gold standards of multiphase CT or MRI. The use of CEUS offers an alternative and reliable modality to diagnose HCC. CEUS is less expensive, it does not involve radiation, does not affect the kidneys, and it allows for multiple doses in one setting. In a meta-analysis, CEUS has been shown to have a sensitivity of 85% and specificity of 91% in diagnosing HCC [12]. Quaia et al., has shown increased sensitivity when CEUS is used in conjunction with CT (97%) as compared with CT only (71–74%) in patients at risk for malignancy secondary to cirrhosis [21]. Our case series supports this finding, although it argues that, at least in some cases, CEUS may be more predictive than CT and if discordant LI-RADS results occur, a tie-breaker should be considered, such as biopsy if amenable, or MRI.

The ACR has created the LI-RADS criteria for noninvasive imaging diagnosis of HCC in patients at risk for its development. Recent articles have reviewed approaches to diagnosing HCC with CEUS [20,22,23]. Additional societies worldwide also have guidelines for noninvasive diagnosis of HCC, including European Federation of Societies for Ultrasound in Medicine and Biology (EFSUMB), World Federation for Ultrasound in Medicine and Biology (WFUMB), and Erlanger Synopsis of Contrast-Enhanced Ultrasound for Liver Lesion Assessment in Patients at risk (ESCULAP), with studies comparing and contrasting them with LI-RADS. Schellhass et al. recently completed a multicenter study comparing prospective diagnosis at the time of CEUS with retrospective categorizing lesions via ESCULAP and LIRADS guidelines [24]. This re-demonstrated accuracy of CEUS for noninvasive diagnosis of HCC and intrahepatic cholangiocarcinoma in cirrhotic patients and found that real-time diagnosis and ESCULAP had highest sensitivities, while LIRADS had superior sensitivity. Negative predictive value of LIRADS was inferior, with positive predictive value similarly high among all three diagnostic categories [24]

Within LI-RADS guidance, there is an additional category for malignant lesion that is not specific for HCC- LI-RADS M [18,19]. CEUS can provide crucial information that can be used to differentiate HCC from intrahepatic cholangiocarcinoma (ICC), by demonstrating differences in their enhancement patterns [23]. As discussed, HCC classically shows arterial hyperenhancement and delayed, mild contrast washout in the late phase [22]. In contrast, ICC shows peripheral arterial contrast enhancement with early contrast washout of the vascularized parts of the lesion in the portal-venous and late phase [23,25]. This may allow for key differentiation between two malignancies and further argues for the usefulness of CEUS as a diagnostic modality. However, it is unclear how common a mixed subtype ICC/HCC is in cirrhotic patients or its enhancement patterns, and biopsy may still be necessary.

Small liver nodules can be a challenge to diagnose in these patients regardless of modality used, and this is no different in CEUS. A recent study by Huang et al. has shown that when CEUS is used for lesions less than 2 cm, that LI-RADS has a high specificity for HCC. It did also note that LI-RADS had lower sensitivity than when guidelines from EFSUMB and WFUMB were utilized [26]. It may be a challenge to accurately determine the LI-RADS categorization of lesions less than 1 cm via morphology due to their small size and this may be more dependent upon the patients’ sonographic characteristics (such as obesity, steatosis, location of the lesion). Regardless, sub-centimeter lesions do not reach noninvasive imaging criteria for HCC diagnosis in LI-RADS, the most they can reach is intermediate (LI-RADS 3) or “probably” HCC (LI-RADS 4) when utilizing CEUS, CT, or MRI. As this does not earn potential transplant patient exception points, nor exclude them from transplant, many institutions will continue to observe these small lesions with shorter interval follow-ups, and treat them when they are larger and definitively declare themselves if growth is observed [16,20].

Adenomas are liver lesions that are rarely seen in cirrhotics, but do in general have a transformation risk to HCC of 5-10%, with risk factors of male sex, glycogen storage disease, anabolic steroid use, and beta-catenin adenoma subtype [27,28]. On imaging, adenomas can show enhancement features that overlap with HCC, and unfortunately there are not yet definitive noninvasive diagnostic characteristics of the highest-risk subtype of beta-catenin on imaging [28]. While different subtypes of adenomas have differing transformation risks, differentiating imaging characteristics are not necessarily dependable, and biopsy may be required to determine the subtype and therefore the transformation risk, or if transformation has already occurred. On CEUS, there is significant overlap of adenoma with HCC in their enhancement patterns [15,22] but there may be differentiation by very early features in how the lesion enhances [15]. It is important to note that CEUS may show washout in adenomas, when MRI does not, which may be attributable to the contrast characteristics—CEUS contrast agents remain intravascular, whereas MRI agents diffuse into the interstitium [28]. Diagnosis in these patients depends upon patient characteristics and cirrhotic risks, consideration of multimodality imaging, and ultimately may depend upon biopsy.

CEUS does have inherent limitations, many of which are shared with routine US. Accuracy depends on the operator with room for variability in interpretation and diagnosis [24]. CEUS also performs similarly to grayscale US where significant adiposity or advanced hepatic steatosis can cause limited visibility [13]. Furthermore, it may not detect very deep lesions (classically dome and central/medial lesions) that are greater than 10 cm from the probe and therefore cannot be characterized via CEUS. However, newer contrast software programs are available that can image significantly greater depths to overcome this limitation. CEUS can target several lesions during a visit and can distinguish portal vein bland versus tumor thrombus [29]. However, screening the whole liver in terms of convenience and cost-effectiveness can prove difficult, as repeated injections of the contrast agent are needed to examine all of the level segments [30]. Moreover, CEUS cannot stage a patient, and single-phase CT may be needed in patients who are at risk for distant HCC metastasis, which would reduce their overall radiation dose.

Future directions with CEUS include continuing studies on accuracy, particularly LIRADS M lesion pathology as these may be a more common finding on CEUS than diagnosed with CT in the cirrhotic population due to continual imaging, as opposed to limited timepoints post contrast in CT. Additional studies have looked at quantitative enhancement patterns and perfusion analysis in HCC [31] by looking at time intensity curves, peak enhancement, and area under the curve to differentiate lesions. However, in the Unites States, not all US contrast software packages currently have this capability. Molecular ultrasound imaging is another emerging field within CEUS, using targeted contrast agents. In the future, this could be in use for diagnosing specific lesions and for targeted therapeutics delivery [32,33].

## 5. Conclusions

Accumulating evidence has demonstrated the important role of CEUS in diagnosing tumors of the liver, and that CEUS can offer certain advantages over or in conjunction with CT in specific patient populations. These include patients with contraindications to CT or MRI due to renal dysfunction, contrast-related allergy, inability to breath-hold without anesthesia, MRI noncompatible pacemaker, and so forth. Our case series shows that, in some cases, the tumors may be visible earlier via CEUS than CT, when both studies are optimized, and when results are discrepant between LIRADS M and LIRADS 5 lesions, biopsy or an alternate tie-breaking imaging modality should strongly be considered. CEUS is an important imaging modality that is complementary to CT for detecting and diagnosing tumors in liver such as HCC and should be incorporated into HCC screening imaging paradigms. Future studies are indicated to evaluate the utility of CEUS for early detection of the subtypes of tumors, and to examine the value of CEUS for monitoring the therapeutic response of HCC.

## Supplementary Materials

The following are available online at https://www.mdpi.com/2305-6320/7/9/51/s1. Video S1: Patient with dynamic arterial phase ultrasound imaging of the liver lesion via after Lumason^®^ injection. Dynamic ultrasound imaging of the right lobe of the liver after contrast administration in Patient 1, during active patient breathing with sweeps through the area of concern. The lesion is seen to enhance above background liver at time stamp 0:24 s and has started to washout by the end of the 60 s clip. Sweeps include images of the right kidney posterior to the liver, and patency of the portal vein.
